# Analysis of the Performance Variation Mechanism of MEMS Suspended Inductors under Mechanical Shock

**DOI:** 10.3390/mi10100686

**Published:** 2019-10-11

**Authors:** Yiyuan Li, Lixin Xu, Jianhua Li

**Affiliations:** School of Mechatronical Engineering, Beijing Institute of Technology, Beijing 100081, China; lyy510@bit.edu.cn (Y.L.); jhli@bit.edu.cn (J.L.)

**Keywords:** MEMS suspended inductor, mechanical shock, MEMS reliability

## Abstract

Micro-electromechanical system (MEMS) suspended inductors have been widely studied in recent decades because of their excellent radio frequency performance. However, the deformation of the inductor coil and the performance variation usually occur to the MEMS suspended inductors when the inductors are under mechanical shock. Few studies have been carried out on the performance variation of MEMS suspended inductors under shock. In this study, the mechanism of the performance variation of MEMS suspended inductors under mechanical shock is analyzed by combining theoretical analysis and experiments. A theoretical analysis based on the lumped-element equivalent model is presented and shock tests are carried out. The shock tests show that the main reason of the MEMS suspended inductor performance variation after mechanical shock is the variation of the substrate parasitic effect, which is caused by the variation of the suspension height of the inductor after shock. The test results agree with the theoretical analysis.

## 1. Introduction

Inductors play an important role in radio frequency integrated circuits (RFICs) [1,2,3]. Compared with traditional complementary metal oxide semiconductor (CMOS) on-chip inductors, micro-electromechanical systems (MEMS) suspended planar spiral inductors have excellent radio-frequency because the inductor coils are lifted several micrometers above the substrate [4,5,6,7]. MEMS suspended inductors have benefits in improving the performance of RFICs. However, MEMS suspended inductors have poor mechanical properties and they tend to fail under mechanical shock during fabrication, shipping, and operation. In particular, in some extreme application conditions, MEMS devices tend to withstand high mechanical shocks of amplitudes in the order of 10^4^–10^5^ g [8]. A suspended structure inductor coil of the MEMS suspended inductor, for example, is susceptible to deform or even damage under high mechanical shock. When the MEMS suspended inductors are shocked and plastic deformation occurs, the radio frequency (RF) performance of the inductors will vary which may affect the operation of the circuits. So, it is important to study the influence of shock on the performance of the MEMS suspended inductors.

Many studies have been carried out to model the MEMS planar spiral inductor on silicon substrate with a lumped-element equivalent circuit [9,10,11], among which the π model presented by Yue et al. [12,13,14] is the most widely used one. Many researchers used the π model to design, optimize, and study MEMS planar spiral inductors. Lee et al. analyzed the effects of the substrate characteristic, metal line width, and suspension height on the suspended inductor performance with the π model [15]. Palan et al. designed and characterized a levitated suspended RF inductor with the π model [16]. Lu et al. designed and optimized a MEMS suspended inductor which was compatibility with the CMOS process with the π model and the inductors they fabricated had a high Q factor [17]. The π model characterizes the planar spiral inductors on silicon with a lumped-element equivalent circuit. The method of calculating each element in the π model by geometric parameters and material parameters is presented in Yue’s work. There are also some researchers who studied to improve the π model. Cao et al. developed a 2-π model for on-chip spiral inductors [18]. An equivalent 2-π ladder circuit were employed to model the series resistance and inductance, the proximity effect and the skin effect of the conductor were considered. The 2-π model can provide an accurate performance prediction and an excellent scalability for spiral inductor design. To obtain the model parameters of an existing MEMS inductor, Huang et al. proposed an approach to extract the values of each element of the π model by using the Y parameters of the inductors obtained by experimental tests [19,20]. The performance of the inductors and the factors affecting the loss can be analyzed with this method. 

In this investigation, the performance variation mechanism of MEMS suspended inductors under mechanical shock is analyzed by combining theoretical analysis and experiments. First, a theoretical analysis is performed based on the π model. Then for the purpose of verification of the theoretical analysis, shock experiments with a Machete hammer are carried out. The values of each π model elements of the inductors shocked by different amplitude shock pulses are extracted. Finally, the experiment results are presented. As the mechanical response analysis and shock test of MEMS suspended inductors have been presented in our former paper [21], we mainly focus on the analysis and discussion of the π model parameters variation and the performance variation mechanism of MEMS suspended inductors under mechanical shock in this paper.

## 2. Theoretical Analysis

**Figure 1:**
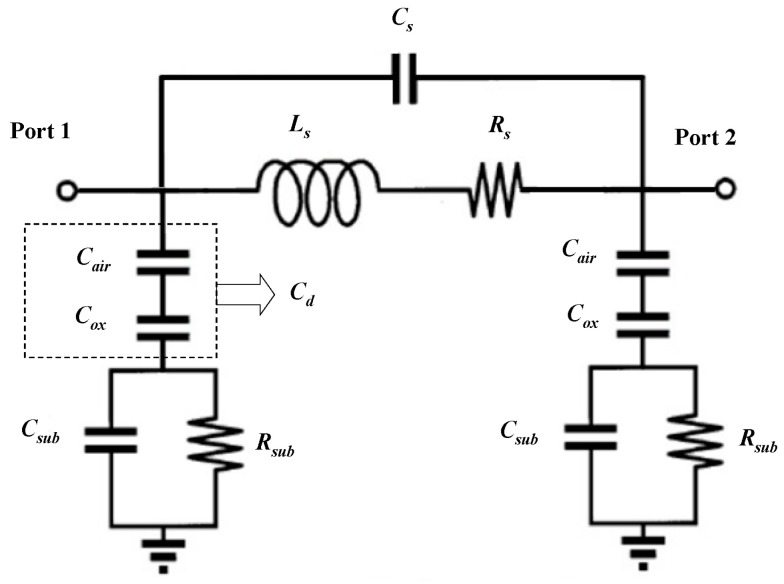
The π model of the micro-electromechanical system (MEMS) suspended inductor.

The RF performance of MEMS suspended inductors can be analyzed with the π lumped-element equivalent model. Figure 1 shows the π model consisting of eleven elements. The equivalent circuit in Figure 1 consists of a series branch and two parallel branches. The series branch characterizes the parameters of the inductor coil and the parallel branch characterizes the parasitic parameters of the silicon substrate.

### 2.1. Series Branch of π Model

The series branch of π model consists of three elements: the series inductance of the inductor coil $L_{s}$, the series resistance of the inductor coil $R_{s}$, and the series capacitance $C_{s}$.

The series inductance $L_{s}$ characterizes the ability of the inductor to store magnetic energy and it can be considered as a constant mainly decided by the geometry of the inductor coil when the wire thickness is thin and the working frequency is high. For the planar spiral inductor coil, the inductance value $L_{s}$ can be calculated with the Greenhouse method [22]. The Greenhouse method calculates the inductance value of the inductor coil by summing the self-inductance of each wire segment and the positive and negative mutual inductance between the wire segment pairs.

The series resistance $R_{s}$ represent the metal loss mechanism which should be expressed as the alternating current (AC) resistance of the inductor coil. Besides the material conductivity and the cross-sectional area of the wire segments, $R_{s}$ is also related to frequency. The AC resistance of the conductor is higher than that of direct current (DC) resistance and the AC resistance increases with frequency because of the existence of skin effect. The series resistance $R_{s}$ can be expressed as [14]:

(1) $$R_{s}=\frac{\rho \cdot l}{w\cdot t_{eff}}$$

where ρ, l, and w represent the resistivity, length, and width of the wire, respectively. $t_{eff}$ represent the effective thickness defining the area of current flowing which can be expressed as Equation (2).

(2) $$t_{eff}=\delta \cdot \left(1-e^{-t/\delta }\right)$$

where t and δ represent the thickness of the wire and the skin depth of the wire material, respectively. The skin depth can be calculated with:

(3) $$\delta =\sqrt{\frac{\rho }{\pi \mu f}}$$

where μ and f represent the permeability of the wire material and the frequency, respectively.

**Figure 2:**
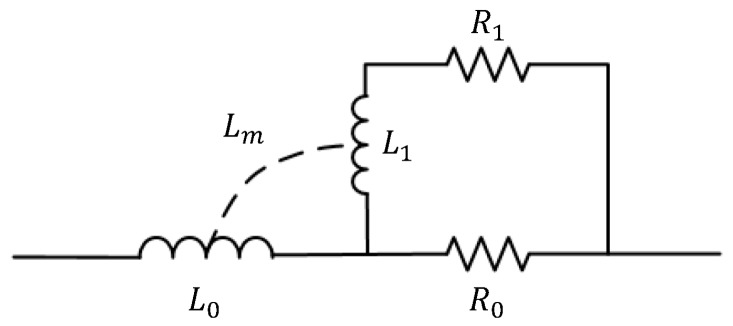
The ladder circuit representing the series resistance and inductance.

According to [18], the series resistance and inductance can also be modeled as a ladder circuit as Figure 2 shows. $R_{0}$ and $L_{0}$ indicated the series resistance and inductance of the wire in DC condition. To take skin effect and proximity effect into account, the additional resistor-inductor branch which includes $R_{1}$ and $L_{1}$ in Figure 2 is introduced to represent each conduction layer in depth and the mutual inductance $L_{m}$ is added to model the magnetic interaction between the external field and internal current. The analytical equation of each parameters in the ladder circuit can be referred to [18].

The series capacitance $C_{s}$ mainly represents the coupling capacitance caused by the overlap between the inductor coil and the underpass because the adjacent turns are almost equipotential and the effect of the crosstalk capacitance is negligible [14]. The wire and the underpass can be considered as the upper and lower plates of the parallel plate capacitor. So, the series capacitance can be expressed as:

(4) $$C_{s}=n\cdot w^{2}\cdot \frac{\varepsilon_{air}}{t_{airM1-M2}}=n\cdot w^{2}\cdot \frac{\varepsilon_{air}}{h-t}$$

where n represents the number of overlap, $\varepsilon_{air}$ represents the permittivity of air, $t_{airM1-M2}$ represent the air layer thickness between the inductor coil and the underpass. $t_{airM1-M2}$ is related to the suspension height h, h indicates the distance between the inductor coil and the upper surface of the insulating layer, the thickness of the underpass line is equal to the coil wire thickness t. $n\cdot w^{2}$ indicates the overlapping area between the coil and the underpass.

### 2.2. Parallel Branch of π Model

Each of the parallel branches of π model consists of three elements: the dielectric layer capacitance $C_{d}$, the substrate parasitic resistance $R_{sub}$, and the substrate parasitic capacitance $C_{sub}$. 

An air layer exists between the inductor coil and the surface of the oxide layer, which is the difference between MEMS suspended inductors and traditional on-chip inductors. So, an air layer capacitance $C_{air}$ is added to the traditional π model and the dielectric layer capacitance $C_{d}$ consists of $C_{air}$ and the oxide capacitance $C_{ox}$ as Figure 1 shows. Considering the inductor coil as the upper plate of the capacitor and the substrate surface as the lower plate of the capacitor, $C_{air}$ and $C_{ox}$ can be calculated with:

(5) $$C_{air}=\frac{1}{2}\cdot l\cdot w\cdot \frac{\varepsilon_{air}}{h}$$

(6) $$C_{ox}=\frac{1}{2}\cdot l\cdot w\cdot \frac{\varepsilon_{ox}}{d_{ox}}$$

where h and $d_{ox}$ represents the suspension height and the oxide layer thickness respectively and $\varepsilon_{ox}$ represents the permittivity of the oxide layer.

A larger area of the inductor coil and a lower suspension height will make the dielectric layer capacitance $C_{d}$ become higher and the capacitance coupling effect of the MEMS suspended inductor become more significant.

$R_{sub}$ and $C_{sub}$ represent the silicon substrate parasitic resistance and capacitance respectively. $C_{sub}$ models the high-frequency capacitive effects occurring in the semiconductor silicon. $R_{sub}$ and $C_{sub}$ can be approximately proportional to the area occupied by the inductor and they can be calculated with Equations (7) and (8) respectively.

(7) $$R_{sub}=\frac{2}{l\cdot w\cdot G_{Si}}$$

(8) $$C_{sub}=\frac{1}{2}\cdot l\cdot w\cdot C_{Si}$$

where $G_{Si}$ and $C_{Si}$ are conductance and capacitance per unit area for the substrate. $G_{Si}$ and $C_{Si}$ depend on the substrate type and the substrate doping, they can be extracted from measurement results.

**Figure 3:**
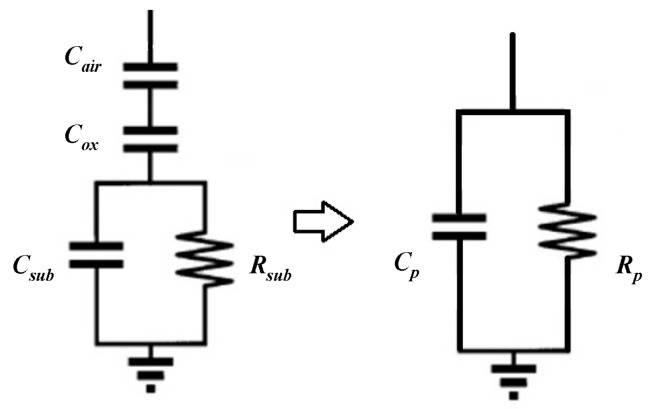
The simplified parallel branch of the π model.

The parallel branch of the π model can be simplified to a resistor–capacitor parallel circuit as Figure 3 shows.

The resistance $R_{p}$ and the capacitance $C_{p}$ represent the substrate loss and the overall substrate parasitic capacitance and they can be expressed as:

(9) $$R_{p}=\frac{1}{\omega^{2}C_{d}{}^{2}R_{sub}}+\frac{R_{sub}{\left(C_{d}+C_{sub}\right)}^{2}}{C_{d}{}^{2}}$$

(10) $$C_{p}=C_{ox}\frac{1+\omega^{2}\left(C_{d}+C_{sub}\right)C_{sub}R_{sub}{}^{2}}{1+\omega^{2}{\left(C_{d}+C_{sub}\right)}^{2}R_{sub}{}^{2}}$$

where $C_{d}$ can be expressed as:

(11) $$C_{d}=\frac{C_{ox}C_{air}}{C_{ox}+C_{air}}.$$

The quality factor Q of the MEMS suspended inductor can be expressed as:

(12) $$Q=2\pi \cdot \frac{\left|E_{M-peak}-E_{E-peak}\right|}{E_{loss}}$$

where $E_{M-peak}$, $E_{E-peak}$, $E_{loss}$ represent the peak magnetic energy, the peak electric energy, and the energy loss in one oscillation n cycle respectively. They can be expressed as:

(13) $$E_{M-peak}=\frac{V_{0}{}^{2}L_{s}}{2\cdot \left(\omega^{2}L_{s}{}^{2}+R_{s}{}^{2}\right)}$$

(14) $$E_{E-peak}=\frac{V_{0}{}^{2}\left(C_{s}+C_{p}\right)}{2}$$

(15) $$E_{loss}=\frac{2\pi }{\omega }\cdot \frac{V_{0}{}^{2}}{2}\cdot \left(\frac{1}{R_{p}}+\frac{R_{s}}{\omega^{2}L_{s}{}^{2}+R_{s}{}^{2}}\right).$$

By substituting Equations (13)–(15) into (12), the quality factor Q can also be expressed as:

(16) $$Q=\frac{\omega \cdot L_{s}}{R_{s}}\cdot \frac{R_{p}}{R_{p}+\left[{\left(\frac{\omega \cdot L_{s}}{R_{s}}\right)}^{2}+1\right]\cdot R_{s}}\cdot \left[1-\frac{R_{s}{}^{2}\left(C_{s}+C_{p}\right)}{L_{s}}-\omega^{2}L_{s}\left(C_{s}+C_{p}\right)\right]$$

where the first term represents the magnetic energy stored and the ohmic loss in the inductor coil, the second term represents the energy dissipated in the silicon substrate which represents the substrate loss and the third term represents the self-resonance factor which describes the decrease of Q as the peak electric energy increases.

As the shock sensitive direction of the suspended inductor is perpendicular to the plane of the coil, the plastic deformation of the suspended inductor is mainly reflected in the variation of the suspension height, which is the distance between the coil and the substrate. The variation of the suspension height leads to the variation of series capacitance $C_{s}$ and the air layer capacitance $C_{air}$. The geometric parameters of the inductor coil i.e., coil length, wire width, and wire thickness will not vary greatly when the suspended inductors are shocked so the series inductance $L_{s}$ and the series resistance $R_{s}$ will not vary greatly. The substrate parasitic resistance $R_{sub}$ and capacitance $C_{sub}$ will also not vary significantly because they are related to the coil length, the wire width of the inductor coil, and the doping of the silicon substrate.

When the suspended inductors are shocked and plastic deformation occurs to them, the suspension height h will decrease. The distance between the plates of the capacitor decreases when the value of h decreases, which will lead to the increase of the capacitance of $C_{s}$ and $C_{air}$ according to the Equations (4) and (5) and the electric energy stored in the inductor will also increase. From Equations (9) and (10), it can be seen that $R_{p}$ will decrease and $C_{p}$ will increase as $C_{d}$ increases. The variation of $R_{p}$ and $C_{p}$ will lead to the increase of the peak electric energy and the energy loss in one oscillation n cycle so the quality factor Q will decrease when the suspended inductor is shocked and plastic deformation is occurred to the inductor coil. From Equation (16), the effect of shock on the quality factor Q variation is reflected in the two terms of substrate loss and self-resonant factor. The smaller the $R_{p}$ is, the more serious the substrate loss is. The higher the $C_{p}$ and $C_{s}$ are, the more the electric energy stored by the suspended inductor is. A lower the Q value indicates a worse performance of the suspended inductor.

## 3. MEMS Suspended Inductors Fabrication and Shock Test

### 3.1. MEMS Suspended Inductor Sample and Fabrication

**Figure 4:**
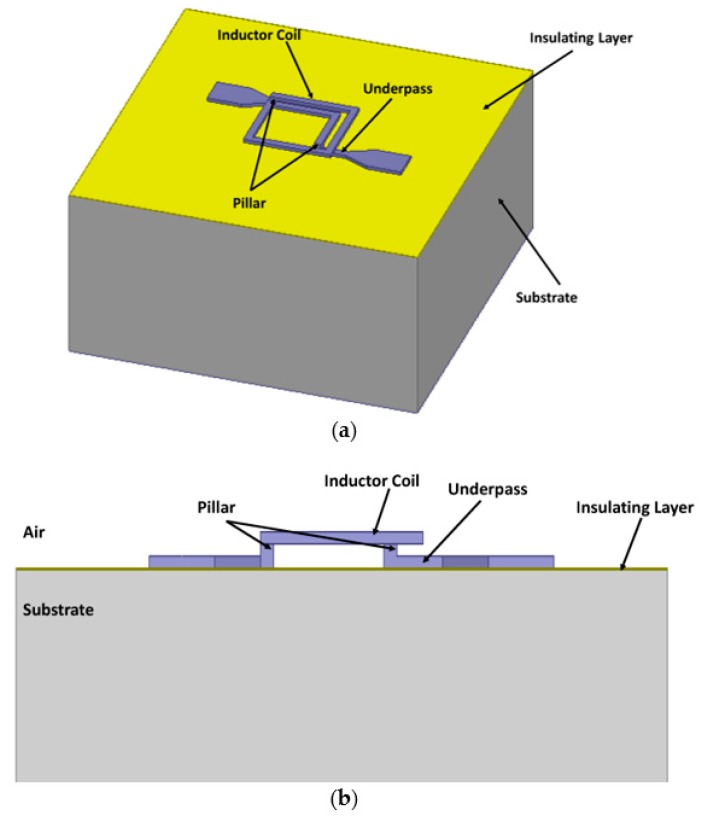
Schematic of the MEMS suspended inductor sample. (**a**) Trimetric view. (**b**) Side view.

**Figure 5:**
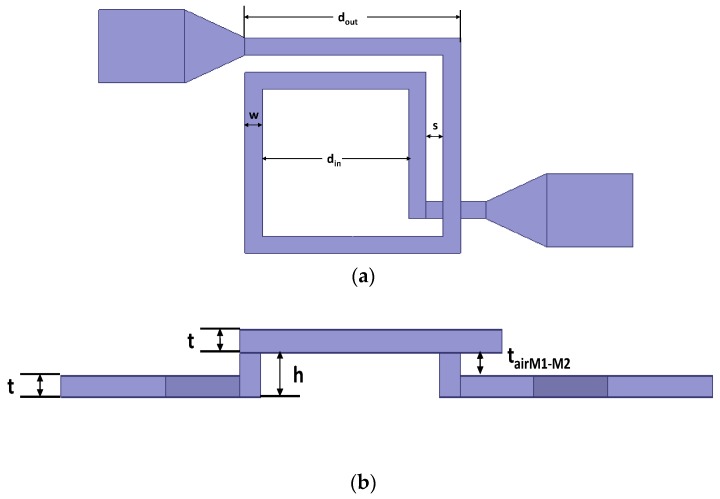
Geometric parameters of the MEMS suspended inductor coil. (**a**) The top view of the inductor coil. (**b**) The side view of the inductor coil.

In this study, we chose the 1.5 turns inductor in [17] as the inductor sample. The schematic of the MEMS suspended inductor is shown in Figure 4 and the geometry parameters of the inductor coil are shown in Figure 5.

In Figure 5, the outer diameter $d_{out}$, inner diameter $d_{in}$, wire width w, spacing s, wire thickness t, and suspension height h are 250 μm, 170 μm, 20 μm, 20 μm, 10 μm, and 20 μm, respectively.

**Figure 6:**
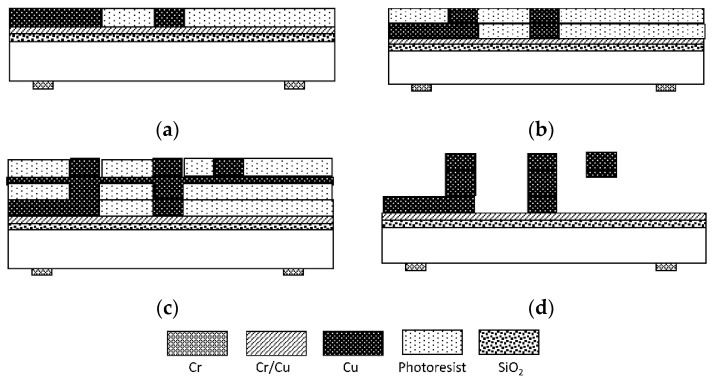
Fabrication process of the MEMS suspended inductor. (**a**) Seed layer deposition, photoresist (PR) mold patterning and copper electroplating, (**b**) PR mold patterning and copper electroplating, (**c**) Seed layer deposition, PR patterning and copper electroplating, (**d**) PR strip and seed layer etch.

The MEMS suspended inductors are fabricated on a silicon wafer and the thickness of the wafer is 0.5 mm. Copper is employed as the material of the inductor and a surface micromachining process based on a positive photoresist is employed to fabricate this MEMS suspended inductor. The fabrication process is shown in Figure 6 and the whole process can be illustrated by the following steps [17]:

1. As Figure 6a shows, a 1.5 μm thick silicon oxide insulating layer is first deposited using a plasma enhanced chemical vapor deposition method. A chromium/copper seed layer is deposited on the silicon oxide by a magnetron sputtering process, the thickness of copper and chromium is 2000 Å and 600 Å, respectively. A 10 μm AZ4620 positive photoresist is spin coated and patterned. Then the underpass lines are electroplated in the molds, electroplating thickness is 10 μm. Copper sulfate is selected as the electroplating bath in all electroplating process steps.
2. As Figure 6b shows, a 10 μm AZ4620 photoresist is spin coated and patterned. Then, the pillars of the suspended inductor are electroplated.
3. As Figure 6c shows, the 1000 Å copper seed layer is deposited and a 10 μm AZ4620 photoresist is spin coated and patterned. Then, the 10 μm spiral coil is finally electroplated.
4. As Figure 6d shows, the suspended structure is released by removing all photoresist and seed layers. Photoresist is removed by sodium hydroxide and acetone, chromium seed layer is removed by mixed solution of potassium ferricyanide and sodium hydroxide, copper seed layer is removed by mixed solution of ammonia and hydrogen peroxide.

**Figure 7:**
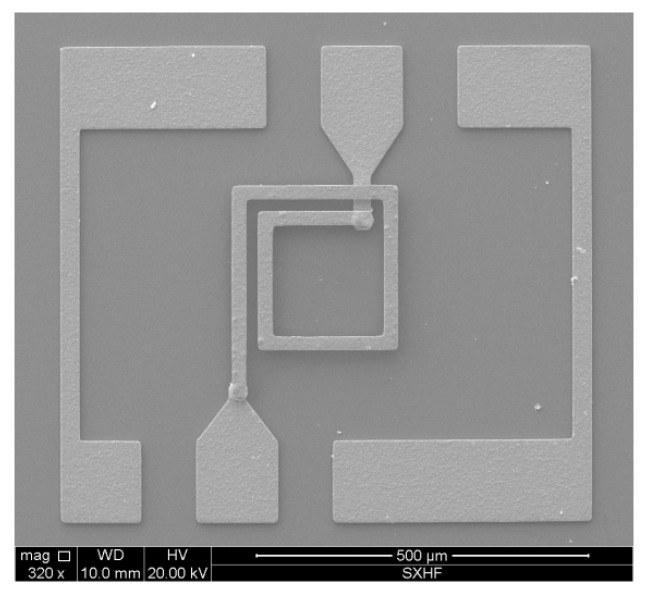
SEM image of the fabricated MEMS suspended inductor.

The scanning electron microscopy (SEM) image of the fabricated MEMS suspended inductor is shown in Figure 7.

The measurement results of the geometric parameters of the suspended inductors are: the outer diameter $d_{out}$ is in the range of 248.6–251.7 μm, the inner diameter $d_{in}$ is in the range of 166.9–171.2 μm, the wire width w is in the range of 20.1–22.8 μm, the spacing s is in the range of 18.3–20.0 μm, the wire thickness is in the range of 7.7–9.1 μm, and the suspension height is in the range of 17.7–18.9 μm. From the measurement results we can find that the consistency of the fabricated inductors is good.

### 3.2. Shock Test

**Figure 8:**
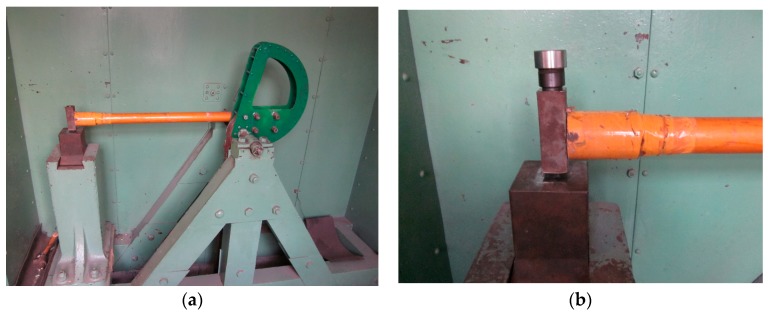
Machete hammer test machine. (**a**) The test machine. (**b**) The hammerhead and the test fixture.

The fabricated MEMS suspended inductors were tested by using a Machete hammer. The Machete hammer test machine is shown in Figure 8. The shock pulses generated by Machete hammer can be considered as half-sine waveforms and the amplitude of the shock pulses generated by the Machete hammer we used range from 8500 to 20,400 g and the durations range from 100 to 120 μs [21]. By changing the lifting height of the hammer, shock loads of different amplitudes can be obtained.

**Figure 9:**
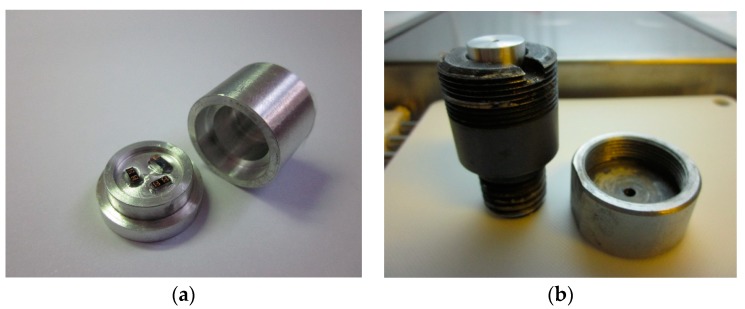
The test shell and the test fixture. (**a**) The shell before assembly. (**b**) The test fixture with the shell.

In order to ensure the consistency of the inductors for the shock test, all inductors for the test were from the same wafer. The wafer with MEMS suspended inductors were divided into several dies and each die had two inductors on it. Three or four dies were adhered to each test shell like Figure 9a shows and the shell was fixed in the test fixture during the shock test like Figure 9b shows.

The normal direction of the coil plane, which is the shock sensitive direction of the inductor, was made parallel to the axial direction of the hammer. The direction of the shock applied was perpendicular to the coil plane.

The MEMS suspended inductors, which were shocked by three kinds of shock loads, were obtained after the shock test. The durations of the three kinds of shock loads were the same and the amplitudes of the three kinds of shock loads were 12,500 g, 13,900 g, and 16,600 g, respectively. Twelve MEMS suspended inductors were employed for each shock test and the shocked inductors were examined with an optical microscope after test. When the shock amplitude was 12,500 g, all of the tested inductors remained intact and no visible damage was found. When the shock amplitude was 13,900 g, no visible damage was found in 7 of the 12 tested inductors. When the shock amplitude was 16,600 g, only one tested inductor remain intact and fracture occurred in 11 of the 12 tested inductors.

## 4. Results and Discussion

**Figure 10:**
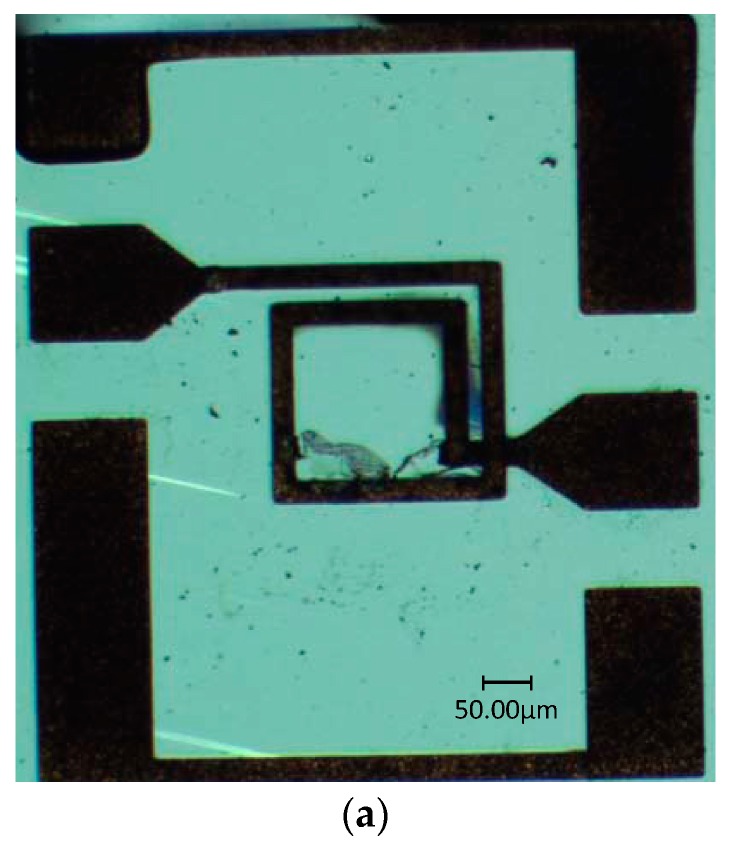

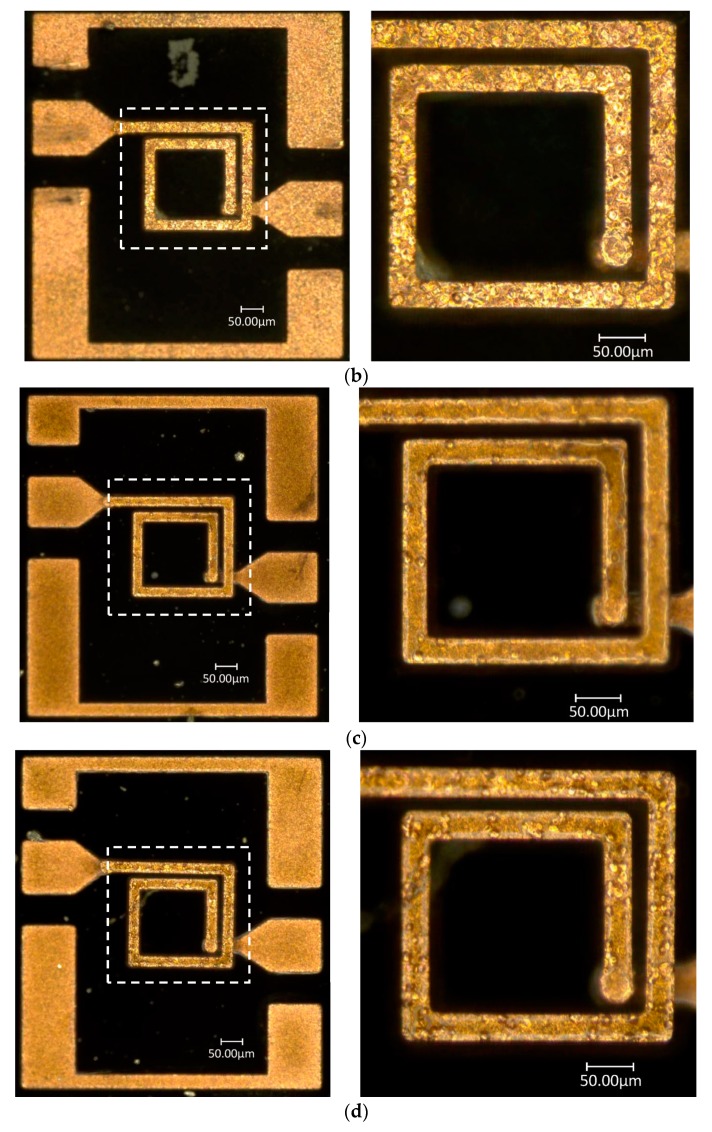
The inductors after shock tests for three shock amplitudes. (**a**) Before shock test. (**b**) 12,500 g. (**c**) 13,900 g. (**d**) 16,600 g.

The performance of four inductors was measured, one of which was an unshocked inductor and the other three were the inductors shocked by three kinds of shock loads with amplitudes of 12,500 g, 13,900 g, and 16,600 g, respectively. The pictures of the three shocked inductors are shown in Figure 10. 

From Figure 10 we can find that plastic deformation occurred to the inductor coils after they were shocked. The inductors after the 13,900 g and 16,600 g shock test had a severer deformation than the inductor after the 12,500 g shock test. We can also find that the inductor coil deforms horizontally besides deforming vertically and the spacing of the coils decreases after shock test from Figure 10, especially for the inductor after the 16,600 g shock test.

With finite element simulation software ANSYS, the variation of the Von Mises equivalent stress and the deformation, which is the decrease of the suspension height, of the inductor coil during shock, can be obtained. The results are listed in Table 1.

**Figure 11:**
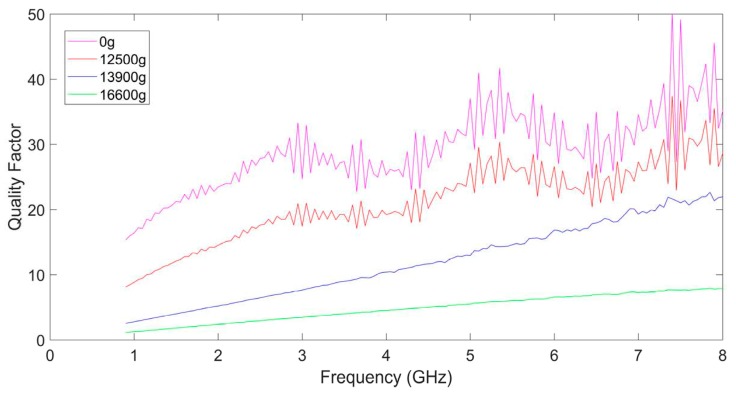
De-embedded quality factors of the inductors.

The S parameters of these MEMS suspended inductors were measured by an Agilent N522A vector network analyzer and a Cascade probe station. The de-embedded quality factors of the four inductors are shown in Figure 11.

As shown in Figure 11, the maximum quality factor of the inductor, which had not been shocked, reached ~40 at 7.5 GHz. When the inductor was shocked by a 12,500 g shock load, the maximum quality factor of the inductor could reach ~30. When the shock amplitude was 13,900 g, the maximum quality factor of the inductor decreased to 23. The maximum quality factor was only 7.8 and the curve became much smoother compared to that of the inductor before the shock test when the shock amplitude reached 16,600 g. From Table 1 we can find that the maximum deformation is about 8 μm when the inductor was subjected to a 16,600 g shock, which means the coil deformed severely and it was close to the underpass and the substrate. The loss brought by high parasitic capacitance is more serious at high frequencies which made the quality factor greatly damped. As the amplitude of the shock load increases, the maximum quality factor of the MEMS suspended inductor decreases, which means the performance of the inductor becomes worse. 

The values of each π model elements: $C_{s}$, $L_{s}$, $R_{s}$, $C_{p}$, and $R_{p}$ can be extracted with the method in [13,19,20] by using the S parameters. 

**Figure 12:**
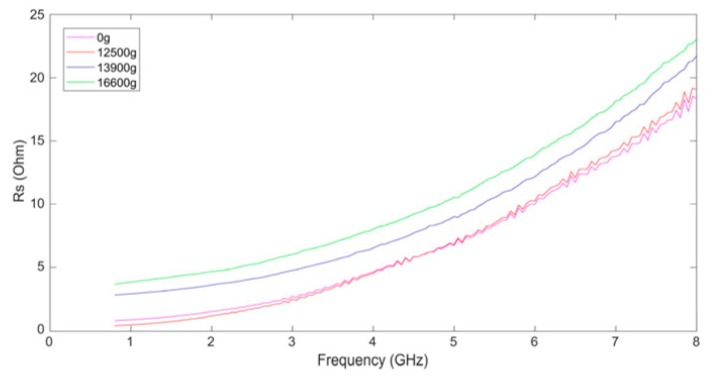
The series resistance of the inductor coils.

Figure 12 shows the series resistance $R_{s}$ of the inductors. From Figure 12 we can find that the series resistance of the inductors increases with the frequency because of the skin effect. The skin effect results in current flowing in the outer area of the wires and the effective cross-sectional area of the wires decreases when the frequency is high. 

It can be found that the $R_{s}$ curve of the inductor shocked by 12,500 g is not greatly varied from that of the unshocked inductor from Figure 12. However, when the shock amplitude rises to 13,900 g and 16,600 g, the series resistance of the inductor coil increases, which means that the mechanical shock also leads to the increase of ohmic loss of the MEMS suspended inductor. We believe that this is because the enhancement of the proximity effect of the wires. The mechanism of the proximity effect is the superposition of the excitation current and the eddy currents, which is caused by the magnetic field generated by the adjacent wire, changes the current distribution in the wires and results in the decrease of the effective cross-sectional area of the wires [18,23]. The proximity effect leads to the increasing of the effective resistance of the inductor wires. From Figure 10, we can find that the inductor coil deforms horizontally besides deforming vertically, which results in the decrease of the spacing between the adjacent wires of the inductor coil. The decrease of spacing will lead to the increase of the eddy currents in wires because the influence of the magnetic field generated by the adjacent wire on them are enhanced, which indicates the enhancement of the proximity effect.

**Figure 13:**
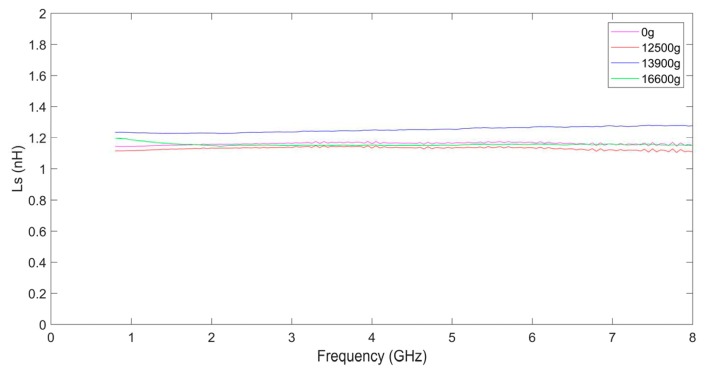
The series inductance of the inductor coils.

Figure 13 shows the series inductance $L_{s}$ of the inductors. From Figure 13 we can find that $L_{s}$ does not vary much with frequency. The series inductance values of the inductors are all in the range of 1.1–1.3 nH and the inductance values of the shocked inductors are not varied greatly from the unshocked inductors. So, the wire width, wire thickness, and length of the coil have not varied greatly after the inductors are shocked.

According to Equation (4) in Section 2.1, the series capacitance $C_{s}$ is related to the suspension height of the coil. So, the series capacitance $C_{s}$ can be predicted and calculated with the simulated deformation of the coil in Table 1. The calculated and the extracted series capacitance are listed in Table 2.

From Table 2 we find that the extracted $C_{s}$ of the unshocked inductor agrees with the calculated $C_{s}$. When the inductor was shocked by 12,500 g, the extracted $C_{s}$ has little variation and it is less than the calculated $C_{s}$. This means that only slight plastic deformation occurred to the inductor coil as the maximum Von Mises stress of the coil (90.31 MPa) is below the yield strength of copper (100 MPa [24]). When the shock amplitude was 13,900 g, the extracted $C_{s}$ increases which means obvious plastic deformation occurred to the coil. The extracted $C_{s}$ is less than the calculated $C_{s}$, we believe that this is because the maximum Von Mises stress (101.16 MPa) is near the yield strength of copper and the actual deformation of the coil is less than the simulated maximum deformation. When the shock amplitude is 16,600 g, the extracted $C_{s}$ agrees with the calculated one which means severe plastic deformation occurs to the coil. As the shock load amplitude of the suspended inductors increases, the series capacitance $C_{s}$ increases, which means that the suspension height of the coil decreases. As the shock amplitude becomes higher, the decrease of the suspension height becomes more significant, which is consistent with the theoretical analysis in Section 2.

**Figure 14:**
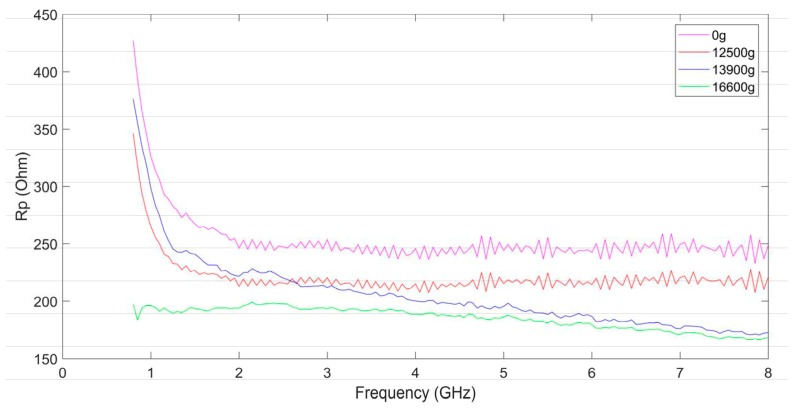
The parasitic resistance of the MEMS suspended inductors.

**Figure 15:**
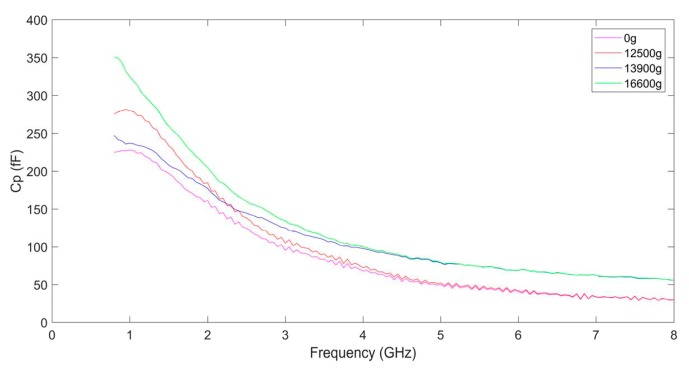
The parasitic capacitance of the MEMS suspended inductors.

Figure 14 and Figure 15 show the parasitic resistance $R_{p}$ and the parasitic capacitance $C_{p}$ in the equivalent model of the suspended inductor. $R_{p}$ and $C_{p}$ represent the substrate loss and the overall substrate parasitic capacitance respectively. They represent the combined effects of $C_{d}$, $C_{sub}$, and $R_{sub}$ in the parallel branches of the π model. $R_{p}$ and $C_{p}$ are frequency depended.

From Figure 14 we can find that the parasitic resistance $R_{p}$ decreases as the frequency increases, which indicates that the influence of the substrate loss on the inductor performance is much more significant at higher frequency. The higher the shock amplitude is, the smaller the parasitic resistance is, which is consistent with the theoretical analysis in Section 2. The decreasing of $R_{p}$ indicates that the substrate loss becomes severer with the increases of the shock amplitude.

From Figure 15 we can find that the parasitic capacitance $C_{p}$ also decreases as the frequency increases, which indicates that the capacitance of the inductor has more significant influence on the inductor performance at lower frequency. The higher the shock amplitude is, the higher the parasitic capacitance is. The electric energy stored by the suspended inductor will be more as the parasitic capacitance is higher. Besides $R_{s}$, $C_{s}$, and $L_{s}$, the parasitic effect of the substrate also has influence on the performance of the MEMS suspended inductor. Although $R_{s}$, $C_{s}$, and $L_{s}$ did not vary much after the inductor shocked by 12,500 g shock load, its Q factor still decreases obviously as the parasitic resistance decreases and the parasitic capacitance increases.

The influence of shock on the performance of the MEMS suspended inductor is reflected in the following aspects: the increase of ohmic loss caused by the increase of the series resistance $R_{s}$, the increase of substrate loss caused by the decrease of the parasitic resistance $R_{p}$, the increase of electric energy stored by the inductor caused by the increase of the series capacitance $C_{s}$ and the parasitic capacitance $C_{p}$.

## 5. Conclusions

In this paper, the mechanism of the performance variation of MEMS suspended inductors under mechanical shock is analyzed by combining theoretical analysis and experiments. First, we theoretically analyzed the variation of the π model parameters caused by the MEMS suspended inductor coil deformation after being shocked. Then MEMS suspended inductors were fabricated and shock tests were carried out. We measured the inductors shocked by shock load with different amplitude and we extracted the π model parameters of the inductors. The variation of the performance and the π model parameters of the inductors before and after shock were analyzed. We find that the quality factor of the inductors decreases in varying degrees after shock. The higher the shock amplitude is, the worse the inductor performance is. The performance variation of the inductors after shock is mainly caused by the variation of the substrate parasitic effect. The loss caused by the increase of the substrate parasitic capacitance and the decrease of the substrate parasitic resistance are the main reasons for the decrease of the quality factor of the MEMS suspended inductor after shock.

## Figures and Tables

**Table 1:** The simulated stress and deformation of the inductor under shock.

Shock Load Amplitude of the Inductor/g	Max Von Mises Stress by Simulation/MPa	Max Deformation by Simulation/μm
0	0	0
12,500	90.31	5.48
13,900	101.16	6.19
16,600	119.94	7.83

**Table 2:** The series capacitance $C_{s}$ of the inductors.

Shock Load Amplitude of the Inductor/g	$\mathrm{The}\;\mathrm{Calculated}\;\mathrm{Series}\;\mathrm{Capacitance}\;C_{s}/\mathrm{fF}$	$\mathrm{The}\;\mathrm{Extracted}\;\mathrm{Series}\;\mathrm{Capacitance}\;C_{s}/\mathrm{fF}$
0	0.42	0.35
12,500	0.84	0.44
13,900	0.97	0.65
16,600	1.72	1.8
